# Multiparametric assessment of atrial cardiopathy in cryptogenic stroke patients: Implications for personalized clinical management

**DOI:** 10.1093/esj/23969873251372773

**Published:** 2026-01-01

**Authors:** Iria López-Dequidt, Sonia Eiras-Penas, Adrián González-Maestro, Carlos Peña-Gil, Emilio Rodríguez-Castro, María Santamaría-Cadavid, José María Prieto-González, José Ramón González-Juanatey, Amparo Martínez-Monzonís

**Affiliations:** Neurology Department, University Clinical Hospital of A Coruña, A Coruña, Galicia, Spain; Cardiology Department, University Clinical Hospital of Santiago de Compostela, Santiago de Compostela, A Coruña, Galicia, Spain; Centre for Biomedical Research Networking Cardiovascular Diseases (CIBERCV), Santiago de Compostela, A Coruña, Galicia, Spain; Cardiology Department, University Clinical Hospital of Santiago de Compostela, Santiago de Compostela, A Coruña, Galicia, Spain; Centre for Biomedical Research Networking Cardiovascular Diseases (CIBERCV), Santiago de Compostela, A Coruña, Galicia, Spain; Cardiology Department, University Clinical Hospital of Santiago de Compostela, Santiago de Compostela, A Coruña, Galicia, Spain; Centre for Biomedical Research Networking Cardiovascular Diseases (CIBERCV), Santiago de Compostela, A Coruña, Galicia, Spain; Neurology Department, University Clinical Hospital of Santiago de Compostela, Santiago de Compostela, A Coruña, Galicia, Spain; Health Research Institute of Santiago de Compostela (IDIS), Santiago de Compostela, A Coruña, Galicia, Spain; Neurology Department, University Clinical Hospital of Santiago de Compostela, Santiago de Compostela, A Coruña, Galicia, Spain; Health Research Institute of Santiago de Compostela (IDIS), Santiago de Compostela, A Coruña, Galicia, Spain; Neurology Department, University Clinical Hospital of Santiago de Compostela, Santiago de Compostela, A Coruña, Galicia, Spain; Health Research Institute of Santiago de Compostela (IDIS), Santiago de Compostela, A Coruña, Galicia, Spain; Cardiology Department, University Clinical Hospital of Santiago de Compostela, Santiago de Compostela, A Coruña, Galicia, Spain; Centre for Biomedical Research Networking Cardiovascular Diseases (CIBERCV), Santiago de Compostela, A Coruña, Galicia, Spain; Cardiology Department, University Clinical Hospital of Santiago de Compostela, Santiago de Compostela, A Coruña, Galicia, Spain; Centre for Biomedical Research Networking Cardiovascular Diseases (CIBERCV), Santiago de Compostela, A Coruña, Galicia, Spain

**Keywords:** Stroke, atrial cardiopathy, atrial fibrillation, left atrial strain, NT-proBNP, Speckle-tracking echocardiography, cryptogenic stroke, multiparametric model, biomarkers, personalized medicine

## Abstract

**Introduction:**

Cryptogenic stroke (CS) represents a heterogeneous group in terms of etiology. Atrial cardiopathy (AC) has emerged as a relevant underlying substrate for both stroke and atrial fibrillation (AF) in these patients. However, no reliable tools are currently available for the early and accurate identification of AC.

**Material and methods:**

We conducted a prospective study including consecutive patients with cardioembolic stroke due to AF (CES-AF), non-cardioembolic stroke (NCES) and cryptogenic stroke (CS). Left atrial strain (LAS) assessed by speckle-tracking echocardiography, and serum markers of AC were evaluated in CES-AF versus NCES patients using ROC curve analysis. Based on these results, we developed a logistic regression model to calculate the probability of AC in CS patients, aiming to discriminate between cardioembolic and non-cardioembolic etiology. Clinical characteristics were compared between CS patients with high (>0.5) and low (<0.5) predicted probability of AC.

**Results:**

A total of 136 patients were included: 44 with CES-AF, 52 with NCES, and 40 with CS. The combination of N-terminal pro-brain natriuretic peptide (NT-proBNP) levels ⩾ 469 pg/mL and biplanar LAS during the contraction phase (LASct) ⩾ -10.2% demonstrated the best-performing AC biomarker combination among those evaluated for identifying cardioembolic etiology (AUC = 0.995). Based on this combination, 30% of CS patients had a predicted probability > 0.5 for AC. These patients were older (77.3 ± 8 vs 68.8 ± 10 years; *p* = 0.011), had more severe strokes (NIHSS score 10.1 ± 7.5 vs 4.6 ± 5.2; *p* = 0.024) and showed a higher incidence of AF during follow-up (6 vs 0 cases; *p* = 0.029).

**Conclusions:**

The combination of NT-proBNP levels and biplanar LASct provides highly sensitive and specific biomarkers of AC. This multiparametric model allows for individualized estimation of AC probability in CS patients, supporting its potential utility in discriminating cardioembolic from non-cardioembolic etiologies and guiding personalized clinical management.

## Graphical Abstract

**Graphical abstract:**
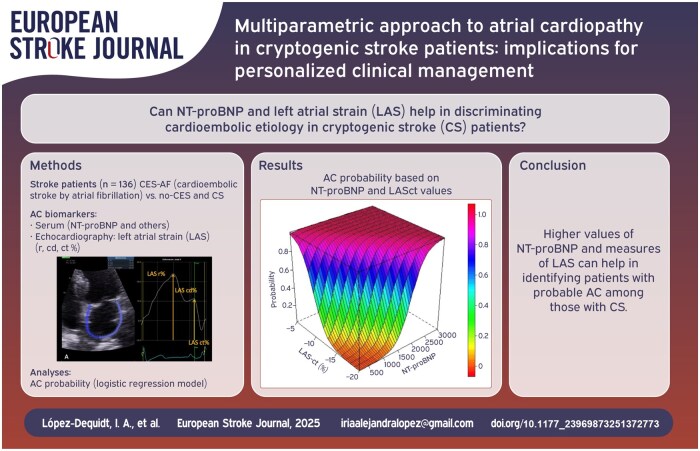

## Introduction

Stroke incidence is currently increasing worldwide, being the second leading cause of death and the third leading cause of combined death and disability, which requires effective prevention strategies.^1^ Ischemic strokes are classified according to their etiology.^2^ When no defined cause is found after a complete stroke work-up, the stroke is classified as cryptogenic (CS); this group accounts for up to 40% of cases, mainly affecting younger patients.^3^ CS represents a heterogeneous group in terms of etiology but frequently originates from cardiac pathology not detected by conventional diagnostic tests. One of the most important causes is atrial cardiopathy (AC), with or without episodes of occult atrial fibrillation (AF).^4^ AC results from tissue remodeling involving inflammation, fibrosis and adipose infiltration, leading to anatomical, electrical, and functional disturbances such as atrial dilatation, arrhythmogenesis and contractile dysfunction. These changes create a thrombogenic substrate that increases the risk of embolic stroke, even in the absence of AF.^5^ For this reason, there is a strong need to identify those CS patients due to AC in order to provide the most appropriate secondary prevention strategy.

Although scientific evidence supports various AC indicators, currently there are no effective and high-precision tools available for its identification in patients with CS. This study was designed to identify the best biomarkers of AC in patients with CES-AF, and subsequently to calculate the probability of AC in each CS patient in order to distinguish a cardioembolic etiology.

## Material and methods

### Study design and participants

A prospective study was conducted by the Cardiology Department in collaboration with the Stroke Unit of the Neurology Department at the University Clinical Hospital of Santiago de Compostela, Spain.

Men and women >55 years were consecutively included if they had one of the following subtypes of ischemic stroke or transient ischemic attack (TIA):

(1) Cardioembolic stroke (CES) due to AF (CES-AF)
(2) Non-cardioembolic stroke (NCES) caused by lacunar or atherothrombotic etiologies
(3) Cryptogenic stroke (CS)

Patients older than 55 years were selected to increase the likelihood of including strokes secondary to AC and to reduce the inclusion of strokes related to patent foramen ovale or other uncommon causes, since age has been shown to be a key factor in the etiology of CS.^6^

Patients were excluded if they met any of the following:

(1) Cardioembolic stroke due to a cause other than AF, as detected by standard diagnostic tests, such as: heart failure with reduced ejection fraction (e.g. dilated cardiomyopathy), left ventricular akinesia, acute myocardial infarction with segmental wall motion abnormalities, cardiac tumor, prosthetic valve thrombosis, or infective endocarditis.
(2) Ischemic stroke due to unusual etiology, including causes other than cardioembolic, lacunar, atherothrombotic and cryptogenic strokes-for example, vasculitis, carotid dissection, carotid web, cerebral vasospasm, drug-induced stroke, or reversible cerebral vasoconstriction syndrome.
(3) Active cancer, except for non-metastatic prostate cancer.

### Stroke assessment

All included patients underwent a comprehensive neurologic assessment including medical history, physical examination, and neuroimaging studies. Brain multimodal computed tomography (CT; non-contrast CT, CT angiography and CT perfusion) or brain magnetic resonance imaging, magnetic resonance angiography, as well as and carotid and transcranial Doppler ultrasonography were performed. Additionally, blood test, electrocardiography (ECG), 24–48 h Holter ECG monitoring, transthoracic echocardiography (TTE) and transesophageal echocardiography were carried out.

The following variables were evaluated in relation to the index stroke:

1. Etiological profile, classified according to the TOAST classification^2^: CES-AF, NCES (atherothrombotic or lacunar) and CS. Atherothrombotic stroke was defined by clinical and imaging findings of significant (>50%) stenosis in an intracranial or extracranial artery.
2. Stroke severity, evaluated using the National Institute Health Stroke Scale (NIHSS) at admission.
3. Demographic characteristics and vascular risk factors, including age, sex, weight, height, arterial hypertension, type 2 diabetes mellitus, dyslipidemia, smoking status, alcohol abuse, and body mass index in accordance with standard guidelines.^7^
4. Acute phase reperfusion therapies, including intravenous thrombolysis and/or mechanical thrombectomy.

### Transthoracic echocardiographic parameters

Cardiac chamber quantification was performed by TTE, and the following parameters were measured according to the recommendations of the European Association of Cardiovascular Imaging and the American Society of Echocardiography (ASE)^8,9^ : left ventricular (LV) end-diastolic and end-systolic diameters (mm), septal and posterior wall thickness (mm), LV end-diastolic volume index (mL/m^2^) and LV end-systolic volume index (mL/m^2^ ), LV mass index (g/m^2^), LV ejection fraction (EF) using the modified Simpson’s biplane method (%), LA anteroposterior diameter (mm), averaged LA area from 2-and 4-chamber views (cm^2^), LA volume index (mL/m^2^), early transmitral flow velocity (E wave, cm/s), late transmitral flow velocity (A wave, cm/s),early diastolic mitral annular velocities at septal (E′) wave and lateral (E′) walls (cm/s), E/E′ratio, E/A ratio, and E-wave deceleration time (s).

#### Left atrial strain (LAS) analysis by speckle-tracking echocardiography

Left atrial deformation imaging was performed using two-dimensional (2D) speckle-tracking echocardiography to assess global longitudinal strain (GLS), following the consensus recommendations published in 2018 by the European Association of Cardiovascular Imaging, the American Society of Echocardiography (ASE) and the Industry Task Force to Standardize Deformation Imaging.^10^

LAS parameters were obtained using dedicated software (EchoPAC version 4.1, General Electric) which allows for semi-automatic quantification of atrial GLS in the 4-chamber and 2-chamber views, as well as a biplanar average.

**Image 1.**
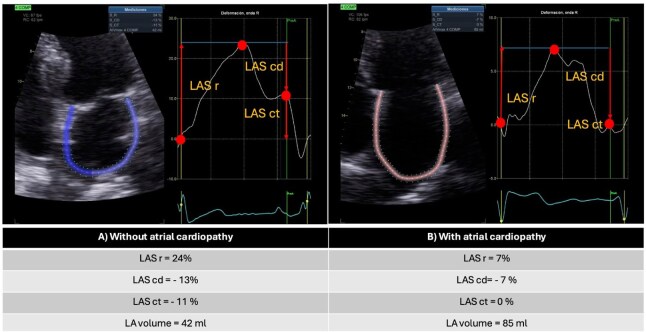
A. Measurement of global longitudinal left atrial strain (LAS) using 2D speckle-tracking in a patient without atrial cardiopathy (AC) performed with dedicated GE software. The measurement points for the different LAS phases are indicated: reservoir phase (r) (%), conduit phase (cd) (%), and atrial contraction phase (ct) (%). B. Measurement of global longitudinal LAS in a patient with AC, in atrial fibrillation rhythm, performed with the same speckle-tracking technique and software. The phases are indicated as above.

The following LA strain components were measured:

- LASr = strain during reservoir phase (%, positive value)
- LAScd = strain during conduit phase (%, negative value)
- LASct = strain during contraction phase (%, negative value; see Image 1).

### Laboratory analysis

Standard biochemical parameters were measured within the first 24 h after the admission using fasting blood samples. These included glucose, glycated hemoglobin (HbA1c %), lipid profile [total cholesterol (mg/dL), high-density lipoprotein (HDL, mg/dL), low-density lipoprotein (LDL, mg/dL), lipoprotein(a) [Lp(a), mg/dL], triglycerides (TGL, mg/dL), uric acid (mg/dL), creatinine (mg/dL), ultrasensitive C-reactive protein (us-CRP, mg/dL) and N-terminal pro-brain natriuretic peptide (NTpro-BNP, pg/mL).

Additionally, fasting blood samples were collected from clinically stable patients in ethylenediaminetetraacetic acid (EDTA) tubes. After centrifugation at 1800 × *g* for 10 min, plasma was aliquoted and stored at −80°C. For further analysis, plasma samples were diluted 1:2 and used to quantify biomarkers associated with AC, selected based on their pathological mechanisms:

a) Atrial stretch: atrial natriuretic peptide (ANP)^11,12^
b) Adiposity: fatty acid binding protein (FABP4) and leptin^13,14^
c) Cytokine-induced stress: growth differentiation factor 15 (GDF15)^15,16^
d) Complement activation: complement component C5a^17,18^
e) Extracellular matrix remodeling: Thrombospondin-2 (TSP-2)^19,20^

Biomarker levels were measured using a magnetic Luminex multiplex test kit (R&D Systems, MN, USA) and expressed in pg/mL or ng/mL. Each sample was analyzed in duplicate. Assays were performed according to the manufacturer’s instructions, with all plasma samples diluted 1:2. Biomarkers were selected based on compatibility with multiplex detection and expected plasma concentrations.

### Assessment of occult AF

Patients with CS underwent 4-week electrocardiographic monitoring using the Nuubo^®^ wearable ECG system to detect occult AF episodes.

### Follow-up for stroke recurrence and new onset AF

Patients with CS were followed for 12 months after the index event to assess for ischemic stroke recurrence and the appearance of new-onset AF.

### Statistical analysis

Continuous variables were expressed as mean ± standard deviation and categorical variables as percentages. Patients with CES-AF were used as a clinical reference model or surrogate gold standard for identifying AC. Clinical, biochemical, and imaging characteristics were compared between CES-AF and NCES patients using the Wilcoxon test for continuous variables and the Fisher’s exact test for categorical variables. A significance level of α = 0.05 was considered. After identifying the structural, functional and biochemical parameters that significantly differed between groups, univariate receiver operating characteristic (ROC) curves were computed to determine the best predictors of AC in the CES-AF group. Based on these, a logistic regression model was developed to calculate the individual probability of AC and discriminate a cardioembolic etiology in CS patients:



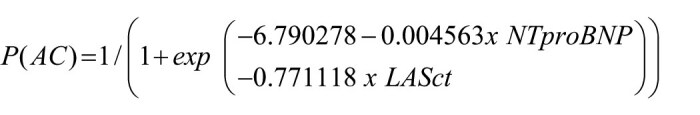



This model was then applied to the CS group. Patients were classified into two groups: High probability of AC if P (AC) > 0.5, and low probability if P (AC) < 0.5. Baseline clinical and demographic characteristics were then compared between these subgroups.

Assuming a conservative and clinically meaningful difference of 600 pg/mL between groups and using a two-tailed α of 0.05 and a power of 90%, the required sample size was estimated to range between 135 and 165 patients.

### Bias control measures

To minimize bias, the following strategies were employed:

1) Echocardiographic measurements were performed by an experienced echocardiographer blinded to stroke subtype classification.
2) Classification of high probability of AC was performed without knowledge of AF detection during the follow-up, which was only assessed after the 12-month period.
3) Since the analysis was based on TOAST classification patients with more than one etiology were not included in the study. Specifically, CES due to causes other than AF, and strokes attributed to two or more possible causes were excluded, as these fall under the “undetermined etiology due to ⩾2 causes category in TOAST .”

## Results

A total of 149 consecutive patients with an ischemic stroke or TIA were evaluated between June 2023 and June 2024. Thirteen patients initially classified as CES-AF were excluded after TTE identified another potential cardioembolic source: nine due to LV akinesia, three due to dilated cardiomyopathy, and one due to infective endocarditis.

The final cohort consisted of 136 patients, distributed as follows: CES-AF = 44; NCES = 52 (atherothrombotic stroke = 24 and lacunar stroke = 28), and CS = 40.

Baseline characteristics of patients with ischemic stroke or TIA, stratified by cardioembolic due to AF (CES-AF) and non-cardioembolic (NCES; lacunar and atherothrombotic) etiology, are summarized in Table 1. Compared to NCES, those with CES-AF were older, more often female, had more severe strokes and more often received mechanical thrombectomy as reperfusion therapy. Notably, there were no significant differences in the prevalence of major vascular risk factors such as hypertension, type 2 diabetes mellitus or dyslipidemia.

**Table 1.** Comparison of clinical, echocardiographic, and laboratory characteristics between CES-AF and NCES patients:.

Variable	CES-AF (*n* = 44)	NCES (*n* = 52)	*p*
Demographic variables			
Age (years)	78.4 ± 9.7	70.9 ± 8.3	<0.001
Women (%)	59	19.2	<0.001
Clinical variables			
NIHSS score	9.8 ± 7.5	4.2 ± 5.2	<0.001
Infarct volume (mL)	24.1 ± 41.4	5.9 ± 14.6	0.001
Intravenous thrombolysis (%)	31.8	11.5	0.084
Mechanical thrombectomy (%)	27.3	7.7	0.013
Vascular risk factors			
Hypertension (%)	81.8	75	0.466
Type 2 diabetes (%)	29.5	44.2	0.204
Dyslipidemia (%)	61.4	76.9	0.120
Smoking (%)	4.7	21.2	0.033
Alcohol abuse (%)	18.6	38.4	0.043
BMI	27.4 ± 3.7	27.9 ± 3.6	0.689
LA analysis by transthoracic echocardiography			
LA diameter (mm)	44.1 ± 8	35.6 ± 4	<0.001
LA area (cm^2^)	27.1 ± 5	17.5 ± 3	<0.001
LA volume (mL/m^2^)	53.8 ± 21	26.9 ± 7.4	<0.001
LA strain analysis by speckle-tracking echocardiography			
LAS r (%)	10.7 ± 6.3	27.7 ± 5.9	<0.001
LAS cd (%)	−8.1 ± 3.8	−9.4 ± 4.7	0.167
LAS ct (%)	−3.0 ± 4.3	−18.2 ± 4.3	<0.001
LA EF (%)	27.5 ± 12.5	57.7 ± 8.3	<0.001
Laboratory analysis			
Glucose (mg/dL)	104.3 ± 24.1	131.9 ± 60.5	0.007
HbA1c (%)	6.0 ± 0.7	6.5 ± 1.5	0.668
Cholesterol (mg/dL)	156.8 ± 32	171.5 ± 40	0.049
LDL (mg/dL)	94.6 ± 29	103.1 ± 32	0.123
HDL (mg/dL)	42.5 ± 11	37.8 ± 9	0.042
Lp (a) (mg/dL)	55.6 ± 642	101.3 ± 100.5	0.013
TGL (mg/dL)	100.1 ± 33	150.1 ± 107.4	<0.001
Us-CRP (mg/dL)	2.8 ± 4	1.3 ± 2	0.003
NT-proBNP (pg/mL)	3089 ± 3899	160 ± 209	<0.001
ANP (pg/mL)	24.6 ± 17.5	7.2 ± 4.6	<0.001
FABP (pg/mL)	32.2 ± 30.1	25.6 ± 13.8	0.817
Leptin (ng/mL)	44.3 ± 43.2	23.2 ± 19.3	0.048
C5a (ng/mL)	15.6 ± 5.1	14.6 ± 11.5	0.027
GDF15 (ng/mL)	3.2 ± 1.5	2.7 ± 1.8	0.018
Insulin (pg/mL)	0.8 ± 0.5	1.3 ± 1.9	0.056
TSP-2 (ng/mL)	31.6 ± 19.7	21.4 ± 11.8	0.009

AC: atrial cardiopathy; AF: atrial fibrillation; ANP: atrial natriuretic peptide; C5a: complement component 5a; CES-AF: cardioembolic stroke due to AF; FABP4: fatty acid binding protein; GDF-15: growth differentiation factor 15; HDL: high-density lipoprotein; LA: Left atrial; LAEF: left atrial ejection fraction; LAS: left atrial strain; LAS cd: left atrial strain during conduit phase; LAS ct: left atrial strain during contraction phase; LAS r: left atrial strain during reservoir phase; LDL: low-density lipoprotein; Lp (a): lipoprotein a; LV: left ventricular; NCES: non-cardioembolic stroke; NIHSS: National Institute Health Stroke Scale; NT-proBNP: N-terminal-pro-B-type natriuretic peptide; TGL: triglycerides; TIA: transient ischemic attack; TSP-2: thrombospondin-2; us-CRP: ultrasensitive C-Reactive protein.

Patients with CES-AF showed more pronounced structural and functional alterations of the left atrium (LA), as well as significantly higher levels of NT-proBNP and ANP, leptin, C5a, GDF15, and thrombospondin-2.

**Figure 1.**
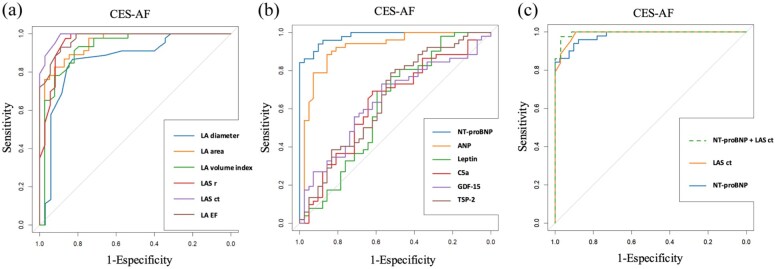
Diagnostic accuracy of echocardiographic (a), serum (b) and combined (c) biomarkers of atrial cardiopathy (AC) for discriminating cardioembolic stroke due to atrial fibrillation (CES-AF), shown as receiver operating characteristic (ROC) curves with corresponding areas under the curve (AUC). (a) Echocardiographic markers of atrial cardiopathy, (b) biochemical markers of atrial cardiopathy, and (c) combined biomarkers of atrial cardiopathy.

Figure 1 displays the ROC curves illustrating sensitivity and specificity for the detection of CES AF using different combinations of echocardiographic and biochemical biomarkers of AC.

The biplanar left atrial strain in contraction (LASct) yielded an area under the curve (AUC) of 0.990, with a cut-off point of −10.2% (sensitivity 100%, specificity 89.2%, positive predictive value (PPV) 100% and negative predictive value (NPV) 91.5%). NT-proBNP levels showed an AUC of 0.98, with a cut-off point of 469 pg/mL (sensitivity 94.1%, specificity 90.2%, PPV 92.5%, and NPV 92.3%).

The combination of NT-proBNP with biplanar LASct values yielded the highest diagnostic performance for CES-AF, with an AUC of 0.995 (sensitivity 97.7%, specificity 97.1%, PPV 97.1%, and NPV 97.7%).

**Figure 2.**
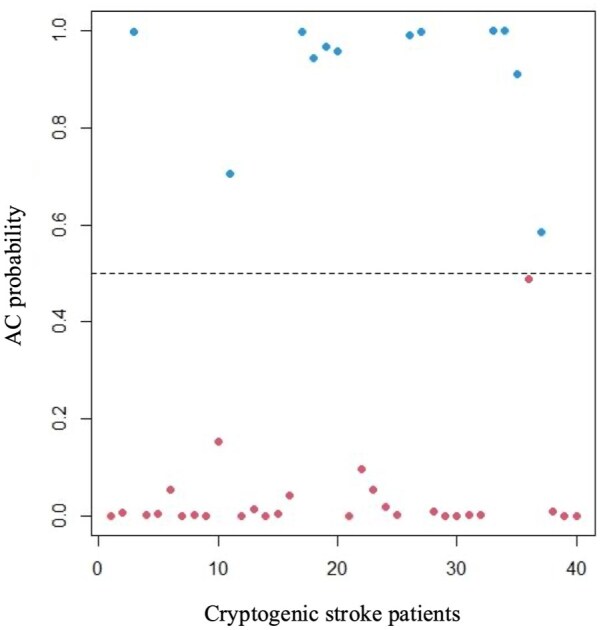
Distribution of individual atrial cardiopathy (AC) probability for each cryptogenic stroke (CS) patient.

Figure 2 illustrate the AC probability for each patient with CS. A total of 30% of CS patients (*n* = 12) had an AC probability > 0.5.

Differences in demographics, stroke characteristics, vascular risk factors, and outcome between CS patients with high (>0.5) and low (<0.5) AC probability are summarized in Table 2. Patients with high AC probability were older (77.3 ± 8 vs 68.8 ± 10 years; *p* = 0.011), presented with more severe strokes (NIH Stroke Scale score:10.1 ± 7.5 vs 4.6 ± 5.2; *p* = 0.024), and a higher incidence of AF during follow-up (6 vs 0; *p* = 0.029). One patient in the high-probability group experienced a recurrent stroke of cryptogenic etiology.

**Table 2.** Characteristics of patients with cryptogenic stroke according to atrial cardiopathy (AC) probability.

Variable	Low AC probability (*n* = 28)	High AC probability (*n* = 12)	*p*
Demographic variables			
Age (years)	68.8 ± 9.7	77.3 ± 8.0	0.011
Women (*n*)	11	10	0.016
Vascular risk factors			
Hypertension (*n*)	25	8	0.168
Type 2 diabetes (*n*)	4	2	1.000
Dyslipidemia (*n*)	21	6	0.154
Smoking (*n*)	5	1	0.648
Alcohol abuse (*n*)	5	2	1.000
BMI (kg/m^2^)	27.8 ± 2.9	30.2 ± 6.6	0.229
Stroke characteristics			
NIHSS (score)	4.6 ± 5.2	10.1 ± 7.5	0.024
Infarct volume (mL)	15.3 ± 20.7	10.6 ± 12.8	0.953
Intravenous thrombolysis (*n*)	5	4	0.416
Mechanical thrombectomy (*n*)	2	3	0.158
Outcome variables			
New onset AF (*n*)	0	6	0.029
TIA or stroke recurrence (*n*)	0	1	0.333

AC: atrial cardiopathy; AF: atrial fibrillation; BMI: Body mass index; NIHSS: National Institute Health Stroke Scale, TIA: transient ischemic attack.

**Figure 3.**
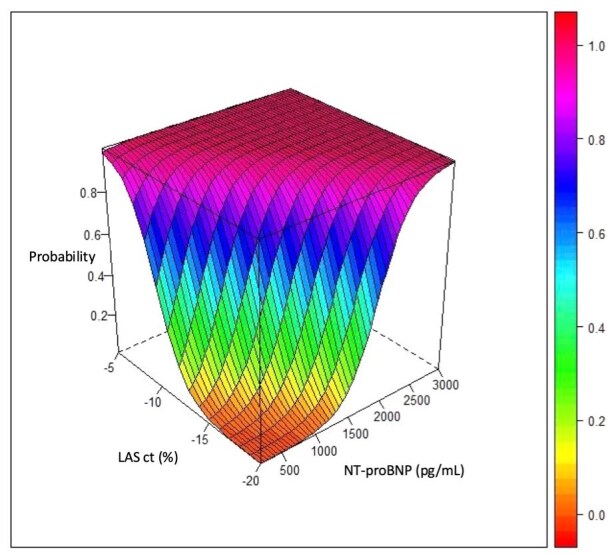
Three-dimensional representation of the probability of atrial cardiopathy (AC) based on NT-proBNP and biplanar LAS-ct values. AC: atrial cardiopathy; NT-proBNP: N-terminal-pro brain natriuretic peptide; LAS-ct: left atrial strain during atrial contraction phase.

Figure 3 depicts the relationship between AC probability and NT-proBNP and biplanar LASct. For any given NT-proBNP value, a LASct > - 5% indicates an AC probability > 95%. Conversely, for any value of LASct < - 20%, NT-proBNP > 2500 pg/mL also correspond to an AC probability > 95%. Similarly, patients with NT-proBNP > 900 pg/mL and LASct > - 10% have a >95% probability of AC, while those with NT-proBNP < 500 pg/mL and LASct < -15% show only residual AC probability (*p* = 0.076).

**Figure 4.**
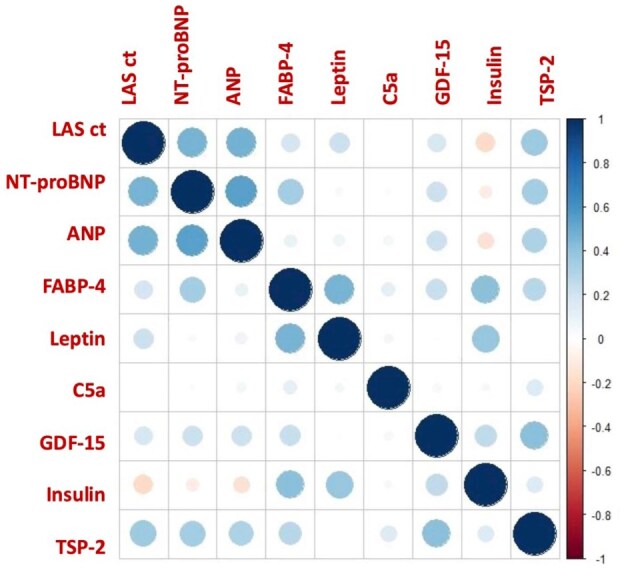
Correlation plot between serum biomarkers and the echocardiographic parameter LASct. Blue indicates positive correlation; red indicates inverse correlation. Color intensity and circle size represent the strength of correlation coefficients (Pearson or Spearman), with larger and more saturated circles indicating stronger associations. ANP: atrial natriuretic peptide; FABP-4: fatty-acid binding protein 4; C5a: complement component 5a;GDF-15: grow differentiation factor 15; LASct: left atrial strain during contraction phase; NT-proBNP: N-terminal pro brain natriuretic peptide; TSP-2: Thrombospondin-2.

Figure 4 displays a correlation plot between the investigated serum markers of AC and the echocardiographic parameter biplanar LASct. A positive correlation was observed between biplanar LASct and NT-proBNP (*r* = 0.76), ANP (*r* = 0.76), and thrombospondin-2 (*r* = 0.31).

## Discussion

This study demonstrates that the combination of plasma NT-proBNP levels (⩾469 pg/mL) together with biplanar LASct (⩾ - 10.2%) obtained through speckle tracking echocardiography, may serve as a reliable imaging-biomarker pair for AC. The AC derived from CES-AF demonstrate high sensitivity and specificity of 97.7% and 97.1% respectively, values not previously reported.

We also developed a novel predictive model using these two parameters to estimate the probability of underlying AC in patients with CS. This tool may aid in identifying patients with possible cardioembolic etiology in absence of AF.

Patients with high probability of AC were older and had more severe strokes, clinical characteristics typically associated with AF-related strokes.^21–23^ These patients also showed a higher incidence of AF during the follow-up. These findings suggest that an underlying AC substrate may contribute to stroke pathogenesis in a subgroup of CS patients.

This study evaluated several plasma biomarkers associated with AC in a stroke population. While markers such as NT-proBNP and ANP have been previously investigated, this is the first study to specifically examine the relationship between these biomarkers and atrial contractile dysfunction in patients with CS. The findings support the potential of biplanar LASct as an imaging marker of AC, showing strong correlations with biomarkers of atrial stretch such as NT-proBNP and ANP. The weaker correlation with thrombospondin-2 may reflect a distinct aspect of atrial remodeling. Thrombospondin-2, a component of the extracellular matrix upregulated in hypertrophic myocardium,^24^ may contribute to a prothrombotic state through platelet activation.^25^ Conversely, the absence of associations between of atrial dysfunction and markers of adiposity or inflammation may reflect a more advanced stage of atrial remodeling in our cohort.

Current diagnosis strategies in CS focus on AF detection through prolonged monitoring.^7^ While this remains important, recent efforts have incorporated clinical, biomarker, and imaging data into predictive models.^26,27^ Speckle tracking parameters such as LASr, LAScd, and LASct have been proposed as predictive imaging markers of incident AF.^28–30^

Emerging evidence suggest that AC itself may represent a pathological substrate for thromboembolism from earlier stages to the development of AF.^31^ However, studies specifically addressing AC in CS are limited. Bhat et al. reported that LASr values > - 23% and LASct values > - 13% discriminated cardioembolic from non-cardioembolic etiology, although with lower diagnostic accuracy than in our model. In addition, these reduced LAS values predicted stroke recurrence.^29^

Our NT-pro-BNP cut-off aligns with previous findings. In a cohort of 264 CS patients, NT-pro-BNP levels were significantly higher in those who developed-AF, with a threshold of ⩾360 pg/ml predicting AF occurrence (OR 5.70, 95% CI 1.11–29.29, *p* = 0.037).^32^ In our cohort, blood samples were collected within 24 h of stroke onset, and evidence suggest that NT-proBNP remains stable during the first week post-stroke,^33^ supporting its clinical applicability in the acute setting.

The findings from our study suggest that LAS measurement using speckle tracking echocardiography offers superior diagnostic utility compared to conventional left atrial size parameters obtained through standard transthoracic echocardiography. This technique is non-invasive, reproducible, and readily applicable in clinical practice, providing more precise insight into atrial mechanical function and underlying atrial cardiopathy.

Unlike other studies, our cohort included only patients aged >55 years. This subgroup represents a higher prevalence of vascular risk factors and a greater likehood of atrial structural changes^34^ However, no significant differences were found in vascular risk profiles between patients with CES-AF and non-CES, or between those classified as in as having a low or high AC probability according to our multiparametric model. These findings reinforce that the combination of LASct and NT-proBNP improves the identification of underlying AC beyond what is achievable with either parameter alone, and independently of traditional vascular risk factors.

The therapeutic implications of AC detection remain uncertain. The ARCADIA trial did not demonstrate benefit of anticoagulation in preventing recurrent stroke among patients with AC. In that trial patients included with >45 years old, and AC was defined by the presence of at least one of the following: P-wave terminal force in ECG lead V1 > 5000 μV• ms, NT-proBNP > 250 pg/mL, or indexed left atrial diameter > 3 cm/m^2^.^35^ In contrast, our study identified LASct and NT-proBNP (⩾469 pg/mL) as more accurate makers of AC. LA diameter showed the lowest diagnostic value in our cohort. Moreover, the biomarkers used in ARCADIA were only moderately predictive of subsequent AF,^36^ suggesting that the selection of more sensitive and specific imaging and biochemical biomarkers, such as those proposed here, may improve AC detection and risk stratification in CS patients.

### Limitations

Our study has several limitations. First, due to the simple size and the prospective design conducted in a single center, our findings should be considered hypothesis-generating and require validation in larger, multicenter clinical trials.

The detection of AC in CS patients through a diagnostic effective tool may support the initiation of appropriate secondary prevention strategies. However, new randomized clinical trials are needed. These trials should ideally focus CS patients identified as having a high probability of AC, evaluating the effect of initiation or withholding a direct oral anticoagulant (DOAC) in the prevention of stroke recurrence.

Another important limitation is related to the use of the TOAST classification to assign stroke etiology. This approach may have included patients with multiple potential causes not fully captured by the TOAST categories, such as non-stenotic (<50%) atherosclerotic plaques in an extracranial or intracranial artery or minor cardioembolic sources.

Moreover, the utility of this multiparametric assessment should be validated in broader populations, including patients both with and without CS.

Finally, although the study included a 1-year retrospective clinical follow-up, no systematic rhythm monitoring was performed. It is important to note, however, that the primary objective was not to identify predictors of occult AF, but rather to characterize atrial cardiopathy as an independent embolic substrate.

## Conclusion

A multiparametric assessment of AC in CS patients, combining plasma NT-proBNP levels with LASct measurements obtained via speckle tracking echocardiography, represents a precise diagnostic tool with high sensitivity and specificity. This method also enables individualized estimation of AC probability in CS patients.

Accurate identification of AC may support the initiation of appropriate secondary prevention strategies, including anticoagulation in those with a high probability of AC. However, prospective clinical trials are needed to confirm its utility and impact on clinical outcomes.

## Abbreviations

AC: atrial cardiopathy

AF: atrial fibrillation

AUC: area under curve

ANP: atrial natriuretic peptide

C5a: complement component 5a

CES-AF: cardioembolic stroke due to AF

CS: cryptogenic stroke

CT: computed tomography

DOAC: direct oral anticoagulants

ECG: electrocardiography

EDTA: ethylenediaminetetraacetic acid

FABP-4: fatty acid-binding protein 4

GDF-15: growth differentiation factor 15

GLS: global longitudinal strain

LA: left atrial

LAS: left atrial strain

LAScd: left atrial strain during conduit phase

LASct: left atrial strain during contraction phase

LASr: left atrial strain during reservoir phase

LV: left ventricular

NCES: non-cardioembolic stroke

NIHSS: National Institute Health Stroke Scale

NT-proBNP: N-terminal pro-brain-natriuretic peptide

TSP-2: Thrombospondin-2

TTE: transthoracic echocardiography

TIA: Transient ischemic attack
